# Associations of Lifestyle Factors with Osteopenia and Osteoporosis in Polish Patients with Inflammatory Bowel Disease

**DOI:** 10.3390/nu13061863

**Published:** 2021-05-30

**Authors:** Anna Maria Rychter, Alicja Ewa Ratajczak, Aleksandra Szymczak-Tomczak, Michał Michalak, Piotr Eder, Agnieszka Dobrowolska, Iwona Krela-Kaźmierczak

**Affiliations:** 1 Department of Gastroenterology, Dietetics and Internal Diseases, Poznan University of Medical Sciences, 60-355 Poznan, Poland; alicjaewaratajczak@gmail.com (A.E.R.); aleksandra.szymczak@o2.pl (A.S.-T.); piotr.eder@op.pl (P.E.); agdob@ump.edu.pl (A.D.); krela@op.pl (I.K.-K.); 2 Department of Computer Sciences and Statistics, Poznan University of Medical Sciences, 60-806 Poznan, Poland; michal@ump.edu.pl

**Keywords:** inflammatory bowel disease, osteoporosis, osteopenia, alcohol, physical activity, coffee, smoking, Crohn’s disease, ulcerative colitis, gastrointestinal diseases

## Abstract

Reduced physical activity (PA), smoking, and coffee and alcohol drinking constitute risk factors of osteoporosis in patients with inflammatory bowel disease (IBD). The aim of the study was to measure the bone mineral density (BMD) and frequency of osteopenia and osteoporosis in patients with IBD and their correlation with PA, smoking, coffee, and alcohol. The study group consisted of 208 patients with IBD-103 with Crohn’s disease (CD), 105 suffering from ulcerative colitis (UC). Densitometric measurements were performed using the DXA. All patients completed a questionnaire concerning PA, smoking, and coffee and alcohol consumption. The prevalence of osteopenia and osteoporosis (L2–L4) in the IBD group was 48.1%; in the CD group, it amounted to 48.6%, and in the UC group, the prevalence was equal to 33.3%. Patients with CD who were diagnosed with osteopenia and osteoporosis demonstrated reduced PA compared to patients with a normal BMD who exercised regularly (*p* = 0.0335). A similar observation was made in the group of women with IBD. Women with a normal BMD exercised significantly more often than women suffering from osteopenia and osteoporosis (*p* = 0.0146). However, no differences in BMD were observed with regard to coffee use, alcohol consumption, or smoking. Thus, since the incidence of osteoporosis in IBD patients is high, it may be dependent on PA.

## 1. Introduction

**Figure 1:**
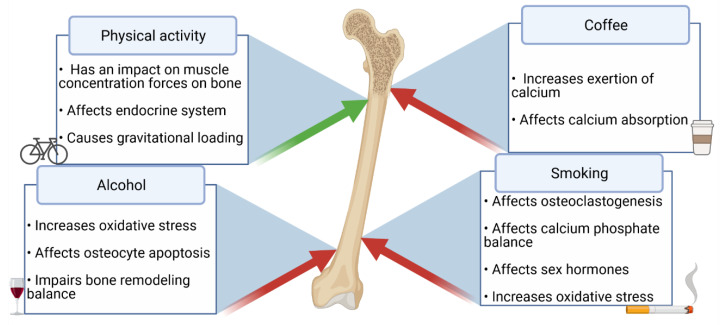
The impact of coffee, alcohol, smoking, and physical activity on BMD and the risk of osteoporosis.

Patients suffering from inflammatory bowel disease (IBD), including Crohn’s disease (CD) and ulcerative colitis (UC), are a group with a higher risk of osteoporosis development [1]. It is vital to notice that in several studies low bone mineral density (BMD) has been more frequently present in CD patients than in UC patients [2]. Nevertheless, in the study using FRAX (Fracture Risk Assessment Tool), CD patients did not present a higher risk of osteoporotic fractures when compared with patients suffering from UC [3]. The pathogenesis of osteoporosis in IBD is multifactorial—risk factors may be divided into unmodifiable (e.g., genetic factors) and modifiable, including reduced physical activity, cigarette smoking, and coffee and alcohol consumption [4,5]. In fact, products containing caffeine, e.g., coffee, may also affect the bone structure [6]. However, the association between coffee consumption and osteoporosis remains unclear, and the results are inconclusive. It is important to bear in mind that coffee is consumed regularly by around 73% of IBD patients [7]. Furthermore, cigarette smoking decreases BMD [8], increases the risk of CD, and decreases the risk of UC development [9,10], and, in spite of the fact that smoking affects CD negatively, it has a “positive” impact on UC [11]. Nevertheless, patients suffering from UC should be encouraged to cease smoking because of other clinical outcomes. In fact, the prevalence of tobacco smoking decreases among IBD patients [12], in spite of the fact that around 10–15% of UC patients still smoke cigarettes [13]. Moreover, Silvennoinen et al. showed that cigarette smoking has been associated with a low BMD in women [14]. On the other hand, although BMD is also affected by alcohol, its consumption probably does not increase the risk of inflammatory bowel disease development [15]. The number of people consuming alcohol among patients with IBD in the United States of America is similar to the general population; nevertheless, 75% of IBD patients reported gastrointestinal complaints following alcohol consumption [16]. The next modifiable contributor to bone health is physical activity (PA), which is especially important during adolescence [17]. However, IBD patients are less active than healthy individuals. CD patients had a more sedentary lifestyle and spent less time exercising at moderate-vigorous intensity than the control group [18]. The impact of coffee, alcohol, and PA on the bone structure is shown in Figure 1. 

Although the benefits and dangers of smoking, exercise, and the consumption of coffee and alcohol for bone health are generally known, these associations were not widely studied in relation to osteoporosis and inflammatory bowel disease. 

## 2. The Aim of the Study

The study aimed to measure the bone mineral density (BMD) and frequency of osteopenia and osteoporosis in patients with IBD and their correlation with PA, smoking, and coffee and alcohol drinking.

## 3. Patients and Methods

**Figure 2:**
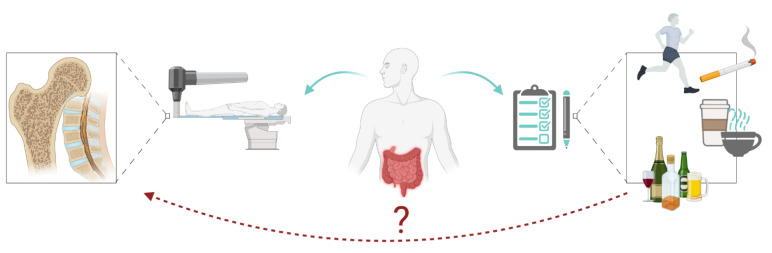
The graphical summary of the methodology.

The study comprised 103 and 105 patients suffering from CD and UC, respectively, who were admitted at the Department of Gastroenterology, Dietetics, and Internal Medicine, Poznan University of Medical Sciences. The study was approved by the Local Bioethics Committee (No. 92/09). Inclusion criteria included written consent for the participation in the research and the diagnosis of CD or UC based on the classical endoscopic, histopathological, and radiological criteria. All patients were treated according to the standards of the Polish Gastroenterology Society and ECCO (European Crohn’s and Colitis Organization). Patients under 18 years of age, pregnant, and patients with diseases that may affect BMD (e.g., diabetes, thyroid disease, celiac disease) were excluded from the study. The graphical summary of the methodology is presented in Figure 2.

### 3.1. Densitometry

Lunar DPX-Plus (using the dual-energy X-ray absorptiometry (DXA)) was used for densitometric measurement—including BMD, T-score, and Z-score—of femoral neck (FN) and lumbar spine (L1–L4). The T-score is the difference between the obtained BMD measurement and mean BMD for young adults, divided by standard deviation for young adults. The Z-score was established as the difference between the measured BMD value and the mean BMD matched by age divided by the standard deviation in the general population. According to the recommendations of the World Health Organization, DXA is the gold standard in the diagnosis of osteoporosis and is used for the BMD classification:

1. Normal: T-score ≥ −1 SD (standard deviation);
2. Osteopenia (low bone mass): T-score <−1 SD and >−2.5 SD;
3. Osteoporosis: T-score ≤ −2.5 SD;
4. Severe osteoporosis: T-score ≤ −2.5 SD with fragility fractures [19].

Additionally, Z-score below −2.5 SD may be indicator of secondary osteoporosis [20].

### 3.2. Lifestyle Factors

All patients completed a questionnaire concerning PA, smoking, and coffee and alcohol consumption. The questions comprised drinking coffee and alcohol (yes/no), smoking (yes/no), and physical activity (low/moderate/high), which were declared by patients, according to their self-assessment. 

### 3.3. Data Analysis

The data were analyzed using the Student’s t-test. If the data did not present the normal distribution, the Mann–Whitney test was applied. The relationship between the categorical data was analyzed by chi-square test for independence. The logistic regression model was applied to check if analyzed demographical and clinical data could constitute a risk factor for osteoporosis. Both univariate and multivariate analysis was performed. The multivariable analysis was performed as stepwise with backward selection. The results were presented as odds ratios (ORs) and their 95% confidence intervals. The analysis was conducted using the statistical software package Statistica PL12 (StatSoft). All tests were two-tailed and were considered statistically significant at *p* < 0.05.

## 4. Results

The percentage of osteoporosis, osteopenia, and a normal bone mineral density occurrence in patients with Crohn’s disease and ulcerative colitis from the lumbar spine region (L2–L4) and femoral neck (FN) is shown in Table 1. Osteopenia was more frequently present than osteoporosis in both UC and CD. Both osteoporosis and osteopenia (L2–L4 and FN) were more frequently present in the CD patients than in patients suffering from UC. The demographic characteristics of the IBD patients included in the study with respect to osteoporosis and osteopenia are presented in Supplementary Table S1.

In terms of the lumbar spine (L2–L4), osteoporosis was not more frequent than osteopenia (*p* = 0.1469), but it occurred more frequently than a normal BMD (*p* = 0.0165). Additionally, there were no significant differences between osteopenia and a normal BMD prevalence (*p* = 0.1115). Therefore, the primary difference stems from the prevalence of osteoporosis and a normal BMD. It is vital to notice that osteoporosis more frequently affects patients with CD than individuals suffering from UC. In our study, CD increased the risk of low BMD, OR= 1.79 (95% CI = [1.03–3.11]), when compared with the UC.

In terms of the FN, there were no significant differences between the prevalence of osteoporosis, osteopenia, and a normal BMD, in comparison with CD and UC. 

In the subsequent statistical analysis, patients were divided according to the T-score index of the lumbar spine region (L2–L4) and the femoral neck (FN) into two groups: I—with a reduced bone mass (osteopenia and osteoporosis), and II—with a normal BMD. The prevalence of a reduced bone mass (osteopenia and osteoporosis) in the IBD group was 48.1% (*n* = 108), whereas the prevalence of a normal bone mass amounted to 51.9% (*n* = 100). The demographic parameters of the IBD patients in the studied group—divided into sex (IBD group), UC, and CD—classified according to a decreased and normal BMD are shown in Table 2 and Table 3. 

CD patients suffering from osteopenia and osteoporosis presented a decreased BMI and body weight than patients with a normal BMD. On the other hand, no differences were observed in weight and BMI among UC patients with a lower and normal BMD, but osteoporotic and osteopenic patients were older than patients with a normal BMD. The differences between IBD groups—female and male subjects—are shown in Table 4 and Table 5. 

Moreover, the risk of low BMD increased with every year of age, OR = 1.03 (95% CI = [1.01–1.05]). The calcium supplementation was associated with enhanced risk of osteopenia and osteoporosis, OR = 3.78 (95% CI = [1.46–9.81]). Higher body mass, height, and BMI significantly decreased the risk of low BMD: OR = 0.96 (95% CI = [0.94–0.98]), OR= 0.95 (95% CI = [0.92–0.98]), and OR= 0.92 (95% CI = [0.86–0.98]), respectively. 

The multivariable analysis showed that age and male sex were the risk factors of low BMD and OR = 18.08 (95% CI = [4.27–76.59]) and OR = 1.05 (95% CI = [1.01–1.10]), respectively. 

No statistically significant differences were found in the frequency of alcohol consumption, coffee drinking, cigarette smoking, and PA between UC patients with a normal and a decreased BMD. However, patients suffering from CD and female IBD patients with a lower BMD were less physically active in comparison with patients with a normal BMD. Table 6, Table 7, Table 8 and Table 9 present detailed information. The lifestyle parameters of the IBD patients with respect to osteoporosis and osteopenia are presented in Supplementary Table S2.

## 5. Discussion

The prevalence of osteopenia and osteoporosis (L2–L4) in the IBD group was 48.1%; in the CD group, it amounted to 48.6%, whereas in the UC group, it was 33.3%. These results are similar to other studies where osteoporosis was present in about 14–42% of patients in the IBD population [21]. In the Swiss IBD cohort study, osteopenia and osteoporosis were found in 57% and 20% of patients, respectively; moreover, CD patients were more likely to suffer from osteoporosis, which is similar to our observations [22]. Furthermore, the authors noticed that the risk of osteoporosis was higher in patients aged ≥50 years when compared with younger patients, although the rates of osteopenia did not differ between the older and younger patients. Our results demonstrate that osteoporotic and osteopenic patients were older than patients with a normal BMD. On the other hand, according to Komaki et al., and their meta-analysis, IBD patients did not present an increased risk of overall fractures, but the risk of spine fractures was significantly elevated and was associated with steroid use [23]. Moreover, Adriani et al. reported that patients with a newly diagnosed IBD presented with a higher risk of osteopenia or osteoporosis [24]. 

Additionally, CD patients with a normal BMD are taller than patients suffering from osteopenia and osteoporosis. It is vital to notice that the growth failure is common in the childhood-onset CD, which may be caused by malnutrition, steroid therapy, and chronic inflammation [25], which also constitute risk factors of osteoporosis [2]. In fact, calcium supplementation increased the odds ratio of low BMD; however, it should be highlighted that it is probably associated with previous imbalance of serum calcium.

Moreover, our study demonstrated that patients with CD who were diagnosed with osteopenia and osteoporosis showed less PA when compared to patients with a normal BMD, who exercised regularly (*p* = 0.0335). A similar observation was made in the group of women with IBD where women with a normal BMD exercised significantly more often than women suffering from osteopenia and osteoporosis (*p* = 0.0146). The study showed that resistance training preserved or improved BMD among the pre- and postmenopausal women [26]. Additionally, total physical activity time protected against osteoporosis in women, whereas leisure physical activity had a protective effect among men [27]. Additionally, physical activity may also be a preventive measure against Crohn’s disease [28]. 

No differences in BMD were observed with regard to coffee, alcohol consumption, or smoking.

Studies regarding coffee consumption, BMD, and the risk of osteoporosis present contradictory results. Moreover, to our knowledge, there are few studies widely investigating this association among the IBD population. In the study by Chang et al., coffee consumption was strongly associated with a higher T-score among men and premenopausal women, which may suggest a protective role of coffee consumption on the osteoporosis risk. Nevertheless, in this study, BMD was assessed by quantitative ultrasound, which is not as precise as DXA [29]. On the other hand, Yang et al. reported that the frequency of osteoporosis was greater among women who did not drink coffee [30], whereas according to the study by Hallström et al., high coffee consumption was associated with a slightly decreased BMD, but it did not increase the risk of fracture [31].

The study by Głąbska et al. showed no significant differences in alcohol consumption between men with IBD in remission and the healthy subjects [32]. Yang et al.’s meta-analysis showed that alcohol consumption was not associated with the elevated risk of Crohn’s disease [33]. On the other hand, in Swanson et al.’s study, the intake of red wine for one week decreased the concentration of calprotectin, a surrogate marker of gastrointestinal inflammation, in the stool of IBD patients [34,35]. Men suffering from alcoholism presented a decreased BMD of the lumbar spine and femoral neck, although this association was not observed in women [36]. Cheraghi et al. reported the association between alcohol consumption and osteoporosis: subjects who consumed one to two and two or more portions of alcohol had a higher risk of osteoporosis by 1.34 and 1.63, respectively, when compared to non-drinkers [37]. On the other hand, in a Korean study, light drinkers, both men and women (consuming alcohol, respectively, 1–19 g/day and 1–9 g/day), demonstrated a higher BMD than non-drinkers and individuals consuming more significant amounts of alcohol [38]. Women, who declared alcohol intake, showed a higher BMD of femur neck than women who did not drink alcohol. There was no significant difference in the lumbar spine BMD [39]. Among men, a higher alcohol intake was associated with a higher risk of hip fracture, i.e., men consuming smaller amounts of alcohol (<14 drinks/week) demonstrated a smaller risk of two or more falls incidents than the abstainers. Conversely, men abusing alcohol had a higher BMD of the femoral neck and the lumbar spine, although simultaneously they demonstrated a higher risk of two or more falls than men who had never been alcoholic [40]. According to a meta-analysis by Berg et al., subjects consuming 0.5–1 portion of alcohol daily presented a smaller risk of hip fracture compared to the subjects with a high alcohol intake and abstinent [41]. 

Clearly, this paper presents interesting and important results, since studies regarding the frequency of osteoporosis and its association with behavioral factors in the IBD populations are scarce. This area seems important as there is a greater awareness of an increased risk of a low BMD among patients with CD or UC, which in turn may be associated with such factors as an increased risk of malnutrition and micronutrient deficiency.

However, one of the main limitations of the study is the use of a not standardized questionnaire regarding the behavioral factors. Although it presents an overall view, it misses both quantitative and qualitative information. Moreover, the amount of essential bioactive substances included in coffee and the exposure to harmful factors are also difficult to assess. Additionally, patients evaluated their physical activity level, which can provide general information regarding PA and BMD, but more specific data are also necessary. Last, but not least, the study group consisted of only 208 individuals, which is not a large group. 

## 6. Conclusions

Osteoporosis constitutes a significant complication of IBD and affects many IBD patients. Several lifestyle factors can affect the risk of osteoporosis due to their impact on BMD. However, the data regarding the consumption of coffee, physical activity, or smoking among IBD patients with BMD assessment are scarce, and if they are present, the research results are inconsistent. In our study, we found a possible protective effect of physical activity on the incidence of osteoporosis among IBD groups. Therefore, PA—adjusted on an individual basis—should be recommended to IBD patients as an essential element of the behavioral treatment. Moreover, further investigations concerning the association of lifestyle factors and the risk of osteoporosis are necessary since they will help implement better preventive and treatment guidelines, particularly in the high-risk group of a decreased BMD, i.e., patients with IBD. 

## Figures and Tables

**Table 1:** Prevalence of osteoporosis, osteopenia, and a normal bone density of the lumbar spine L2-L4 level and the femoral neck, based on T-scores, in patients with Crohn’s disease and ulcerative colitis.

	Category	Osteoporosis *n* (%)	Osteopenia *n* (%)	Normal Bone Mass *n* (%)	*p*-Value
Group					
		L2–L4			
CD		12 (11.7%)	38 (36.9%)	53 (51.5%)	*p* = 0.0269
UC		4 (3.8%)	31 (29.5%)	70 (66.7%)	
		FN			
CD		6 (5.8%)	37 (35.9%)	60 (58.3%)	*p* = 0.1218
UC		3 (2.9%)	27 (25.7%)	75 (71.4%)	

%: Percentage of all patients, CD: Crohn’s disease, UC: ulcerative colitis, L2–L4: Lumbar vertebrae from 2 to 4, FN: Femoral neck.

**Table 2:** The demographic and anthropometric parameters of the studied CD patients with respect to osteoporosis and osteopenia.

Demographic Characteristics	CD	Osteopenia and Osteoporosis	Normal BMD	*p*-Value
N (%)	103	61 (59.2%)	42 (40%)	
Age (Years) (mean ± SD)	35.78 ± 12.78	37.03 ± 13.27	33.95 ± 11.95	*p* = 0.2693
Weight (kg) (mean ± SD)	63.39 ± 13.71	59.2 ± 12	69.48 ± 13.89	*p* = 0.0004
Height (m) (mean ± SD)	171.17 ± 10.19	168.49 ± 9.82	175.07 ± 9.54	*p* = 0.0017

CD- Crohn’s disease; BMD- bone mineral density; BMI – body mass index.

**Table 3:** The demographic and anthropometric parameters of the studied UC patients with respect to osteoporosis and osteopenia.

Demographic Characteristics	UC	Osteopenia and Osteoporosis	Normal BMD	*p*-Value
N (%)	105	48 (45.7%)	57 (54.3%)	
Age (Years) (mean ± SD)	39.56 ± 14.97	44.38 ± 15.62	34.94 ± 12.72	*p* = 0.0036
Weight (kg) (mean ± SD)	68.38 ± 14.83	65.78 ± 15.43	70.06 ± 13.95	*p* = 0.1355
Height (m) (mean ± SD)	171.01 ± 9.8	168.94 ± 10.04	173.69 ± 9.45	*p* = 0.0554
BMI (kg/m^2^) (mean ± SD)	23.29 ± 4.28	22.99 ± 4.78	23.15 ± 3.93	*p* = 0.4451

UC- ulcerative colitis; BMD- bone mineral density; BMI – body mass index.

**Table 4:** The demographic and anthropometric parameters of the studied female IBD patients with respect to osteoporosis and osteopenia.

Demographic Characteristics	All Subjects (IBD) Female	Osteopenia and Osteoporosis	Normal BMD	*p*-Value
N (%)	107	56 (52.3%)	51 (47.7%)	
Age (Years) (mean ± SD)	39.58 ± 14.71	43.91 ± 15.49	34.82 ± 12.29	*p* = 0.0024
Weight (kg) (mean ± SD)	58.73 ± 12.43	55.07 ± 11.17	62.74 ± 12.61	*p* = 0.0019
Height (m) (mean ± SD)	164.08 ± 7.34	161.16 ± 5.42	167.29 ± 7.85	*p* < 0.0001
BMI (kg/m^2^) (mean ± SD)	21.77 ± 4.18	21.2 ± 4.21	22.39 ± 4.09	*p* = 0.1103

IBD- inflammatory bowel disease; BMD- bone mineral density; BMI – body mass index.

**Table 5:** The demographic and anthropometric parameters of the studied male IBD patients with respect to osteoporosis and osteopenia.

Demographic Characteristics	All Subjects (IBD) Male	Osteopenia and Osteoporosis	Normal BMD	*p*-Value
N (%)	101	52	49	
Age (Years) (mean ± SD)	35.68 ± 13.03	36.27 ± 12.88	35.06 ± 13.29	*p* = 0.5338
Weight (kg) (mean ± SD)	73.52 ± 12.49	69.6 ± 12.68	77.68 ± 10.94	*p* = 0.0007
Height (m) (mean ± SD)	178.51 ± 6.37	176.79 ± 6.61	180.35 ± 5.6	*p* = 0.0121
BMI (kg/m^2^) (mean ± SD)	23.08 ± 3.93	22.28 ± 4.07	23.94 ± 3.63	*p* = 0.0174

IBD- inflammatory bowel disease; BMD- bone mineral density; BMI – body mass index.

**Table 6:** The lifestyle parameters of the study CD patients with respect to osteoporosis and osteopenia.

	CD	Osteopenia and Osteoporosis	Normal BMD	*p*-Value
N (%)	103	61 (59.2%)	42 (40%)	
Smoker N (%)	28 (27.2%)	14 (23%)	14 (33.3%)	*p* = 0.2445
Alcohol consumption N (%)	36 (35%)	21 (34.4%)	15 (35.7%)	*p* = 0.8928
Coffee drinker N (%)	57 (55.3%)	33 (54.1%)	24 (57.1%)	*p* = 0.76
Exercise N (%)	66 (64.1%)	34 (55.7%)	32 (76.2%)	*p* = 0.0335

CD- Crohn’s disease; BMD- bone mineral density.

**Table 7:** The lifestyle parameters of the studied UC patients with respect to osteoporosis and osteopenia.

Demographic Characteristics	UC	Osteopenia and Osteoporosis	Normal BMD	*p*-Value
N (%)	105	48 (45.7%)	57 (54.3%)	
Smoker N (%)	8 (7.6%)	3 (6.3%)	5 (8.8%)	*p* = 0.6674
Alcohol consumption N (%)	42 (40%)	19 (39.6%)	23 (40.4%)	*p* = 0.9361
Coffee drinker N (%)	67 (63.8%)	31 (64.6%)	36 (63.2%)	*p* = 0.6801
Exercise N (%)	62 (59%)	27 (56.3%)	35 (61.4%)	*p* = 0.7639

UC- ulcerative colitis; BMD- bone mineral density.

**Table 8:** The lifestyle parameters of the study IBD female patients with respect to osteoporosis and osteopenia.

Demographic Characteristics	All Female Subjects (IBD)	Osteopenia and Osteoporosis	Normal BMD	*p*-Value
N (%)	107	56 (52.3%)	51 (47.7%)	
Smoker N (%)	10 (9.3%)	6 (10.7%)	4 (7.8%)	*p* = 0.6063
Alcohol consumption N (%)	33 (30.8%)	17 (30.4%)	16 (31.4%)	*p* = 0.911
Coffee drinker N (%)	65 (60.7%)	32 (57.1%)	33 (64.7%)	*p* = 0.4214
Exercise N (%)	56 (52.3%)	23 (41.1%)	33 (64.7%)	*p* = 0.0146

IBD- inflammatory bowel disease; BMD- bone mineral density.

**Table 9:** The lifestyle parameters of the studied IBD male patients with respect to osteoporosis and osteopenia.

Demographic Characteristics	All Male Subjects (IBD)	Osteopenia and Osteoporosis	Normal BMD	*p*-Value
N (%)	101	52 (51.5%)	49 (48.5%)	
Smoker N (%)	26 (25.7%)	11 (21.2%)	15 (30.6%)	*p* = 0.2803
Alcohol consumption N (%)	45 (44.6%)	23 (44.2%)	22 (44.9%)	*p* = 0.9436
Coffee drinker N (%)	59 (58.4%)	32 (61.5%)	27 (55.1%)	*p* = 0.5143
Exercise N (%)	72 (71.3%)	38 (73.1%)	34 (69.4%)	*p* = 0.6812

IBD- inflammatory bowel disease; BMD- bone mineral density.

## Data Availability

Data are available in publicly accessible databases. The data presented in this study are openly available in Medline and PubMed databases and on the publisher’s website. The keywords that were used: “osteoporosis”, “osteopenia”, “inflammatory bowel disease”, “diet”, “physical activity”, “coffee”, and “alcohol”. All data in the text are quoted and all works used are listed in the bibliography along with DOI and reference numbers.

## Supplementary Materials

The following are available online at https://www.mdpi.com/article/10.3390/nu13061863/s1, Table S1: Demographic parameters of the studied IBD patients with respect to osteoporosis and osteopenia, Table S2: Lifestyle parameters of the studied IBD patients with respect to osteoporosis and osteopenia.
