# Supplementary material for: Associations of Lifestyle Factors with Osteopenia and Osteoporosis in Polish Patients with Inflammatory Bowel Disease
Source: Nutrients. 2021 May 30;13(6):1863. doi: 10.3390/nu13061863 (PMC8227497; doi:10.3390/nu13061863)
Supplement: Supplementary file 1 [file nutrients-13-01863-s001.zip › nutrients-1216394-supplementary.pdf]

Supplementary Table S1. Demographic characteristic parameters of the study IBD patients stratified by osteoporosis and osteopenia.

Demografic Characteristics	All Subject (IBD)	Osteopenia and Osteoporosis	Normal BMD	p-Value
N (%)	208	108 (48,1%)	100 (51,9%)	
Age (Years)	37,69 ± 14,02	40,23 ± 14,73	34,94 ± 12,72	p = 0,0096
Weight (kg)	65,91 ± 14,47	62,06 ± 13,93	70,06 ± 13,95	p= 0,0001
Hight (m)	171,09 ± 9,97	168,69 ± 9,87	173,69 ± 9,45	p= 0,0004
BMI (kg/m^2)	22,41 ± 4,1	21,72 ± 4,16	23,15 ± 3,93	p= 0,0066

BMI – body mass index; IBD – inflammatory bowel disease; BMD – bone mineral density.

Supplementary Table S2. Lifestyle characteristic parameters of the study IBD patients stratified by osteoporosis and osteopenia.

Demografic Characteristics	All Subject (IBD)	Osteopenia and Osteoporosis	Normal BMD	p-Value
N (%)	208	108 (48,1%)	100 (51,9%)	
Smoker N (%)	36 (17,3 %)	17 (15,7 %)	19 (19 %)	p= 0,5537
Alcohol consumption N (%)	78 (37,5 %)	40 (37 %)	38 (38 %)	p= 0,886
Coffee drinker N (%)	124 (59,6 %)	64 (59,3 %)	60 (60 %)	p= 0,9134
Exercise N (%)	128 (61,5 %)	61 (56,5 %)	67 (67 %)	p= 0,1193

IBD – inflammatory bowel disease; BMD – bone mineral density.
